# Geometric changes in aortic root replacement using Freestyle prosthesis

**DOI:** 10.1186/s13019-021-01583-y

**Published:** 2021-07-28

**Authors:** Anja Osswald, Alina Zubarevich, Arian Arjomandi Rad, Robert Vardanyan, Konstantin Zhigalov, Daniel Wendt, Bastian Schmack, Ahmed Mashhour, Arjang Ruhparwar, Alexander Weymann

**Affiliations:** 1 Department of Thoracic and Cardiovascular Surgery, West-German Heart and Vascular Center Essen, Essen, Germany; 2 Department of Medicine, Faculty of Medicine, Imperial College London, London, UK; 3 Department of Cardiac and Vascular Surgery, Herz-Kreislauf-Zentrum Rotenburg an der Fulda, Rotenburg an der Fulda, Germany

**Keywords:** Aortic valve replacement, Aortic root, Bioroot, Computed tomography measurement

## Abstract

**Background:**

The Medtronic Freestyle prosthesis has proven to be a promising recourse for aortic root replacement in various indications. The present study aims to evaluate clinical outcomes and geometric changes of the aorta after Freestyle implantation.

**Methods:**

Between October 2005 and November 2020, the computed tomography angiography (CTA) data of 32 patients were analyzed in a cohort of 68 patients that underwent aortic root replacement using Freestyle prosthesis. The minimum and maximum diameters and areas of the aortic annulus, aortic root, ascending aorta, and the proximal aortic arch were measured at a plane perpendicular to the long axis of the aorta using 3D multiplanar reconstruction in both the preoperative (*n* = 32) and postoperative (*n* = 10) CTAs. Moreover, volumetric changes of the aortic root and ascending aorta were quantified.

**Results:**

Mean age was 64.6 ± 10.6 years. Indications for surgery using Freestyle prosthesis were combined aortic valve pathologies, aortic aneurysm or dissection, and endocarditis, with concomitant surgery occurring in 28 out of 32 patients. In-hospital mortality was 18.6%.

Preoperative diameter and area measurements of the aortic annulus strongly correlated with the implanted valve size (*p* < 0.001). Bicuspid valve was present in 28.1% of the patients. Diameter and areas of the aortic root decreased after freestyle implantation, resulting in a reduction of the aortic root volume (45.6 ± 26.3 cm^3^ to 18.7 ± 4.5 cm^3^, *p* = 0.029). Volume of the aortic root and the ascending aorta decreased from 137.3 ± 65.2 cm^3^ to 54.5 ± 21.1 cm^3^ after Freestyle implantation (*p* = 0.023).

**Conclusion:**

Implantation of the Freestyle prosthesis presents excellent results in restoring the aortic geometry. Preoperative CTA measurements are beneficial to the surgical procedure and valve selection and therefore, if available, should be considered in pre-operative planning.

## Background

The choice of valve prosthesis in aortic valve and root surgery must be tailored to individual patient needs and characteristics. Medtronic Freestyle prosthesis offers a promising recourse for patients when aortic root replacement becomes necessary. Indications include aortic stenosis or regurgitation in combination with an enlargement of the aortic root or ascending aorta, as well as aortic dissection. Furthermore, a stentless valve is often used in high-risk patients with infective endocarditis of the aortic valve with periannular abscess [1, 2]. Stentless aortic prosthesis provide good clinical outcomes and excellent hemodynamics, particularly in smaller aortic annuli, and further prevent patient-prosthesis mismatch [3, 4].

Echocardiography remains the gold standard for pre- and postoperative diagnosis of aortic valve disease [5]. Since surgical aortic valve sizing is intraoperatively performed using visual observation and measurement, computed tomography is largely rendered unnecessary for sizing measurements. In fact, computed tomography angiography (CTA) is only performed for special indications, typically when echocardiographic findings suggest an aortic pathology in the presence of a bicuspid aortic valve or in the case of minimally invasive surgery [6]. In accordance with guidelines, CT evaluation is helpful in gaining more insight into aortic geometry, improving preoperative planning, and contributes to the evaluation of the severity of the valvular disease [5]. Furthermore, due to aortic root asymmetry, it’s dimensions can be underestimated in echocardiography [7].

In this CTA based study, we assessed the geometry of the aortic valve, root, and ascending aorta before and after the implantation of the Freestyle prosthesis alongside the associated clinical outcomes.

## Methods

### Study design and population

This study is of a retrospective design with prospectively collected data and analysis of CTA images. The study was approved by the local ethics committee and patient consent was waived (21–9907-BO).

**Fig. 1:**
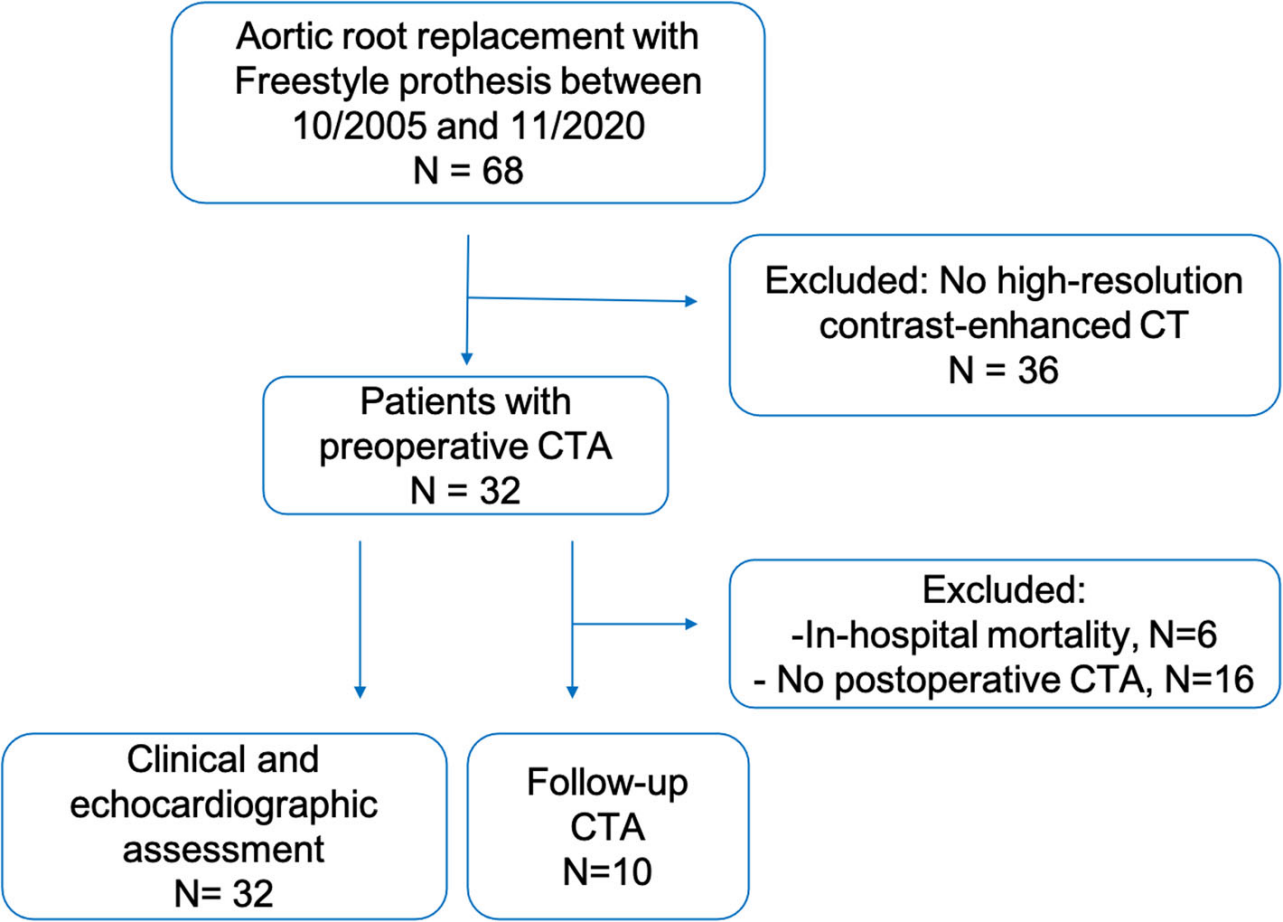
Flowchart of patient inclusion

**Fig. 2:**
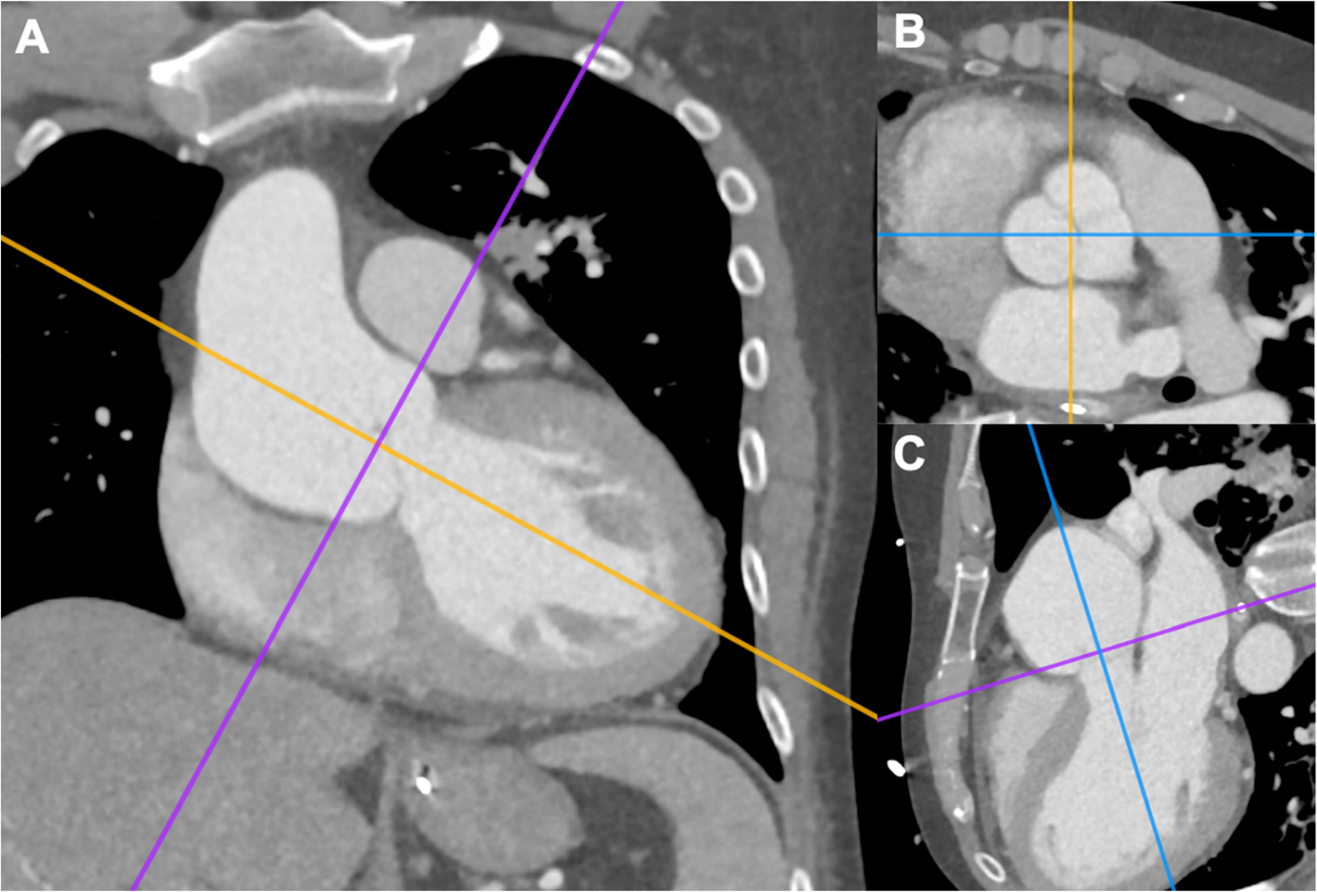
3-dimensional computed tomography reconstruction, with the visualization of the aortic root on a plane perpendicular to the long axis of the aorta (**A**-**C**)

Between October 2005 and November 2020, 68 consecutive patients underwent aortic valve replacement (AVR) using the Medtronic Freestyle prosthesis (Medtronic Inc., Minneapolis, MN) at our institution. Of those, 32 patients received a preoperative CTA, with follow-up CTAs performed in 10 patients, after a median of 18 days [interquartile range (IQR): 12.5–457.0]. Indication for CTAs included ascending aortic aneurysm, aortic dissection, acute chest pain and unknown dyspnea, and previous cardiac surgery. All 32 aformentioned patients were included in this study (Fig. 1). Patient demographics, baseline clinical characteristics, echocardiographic findings, intraoperative parameters, and postoperative outcomes were evaluated. Aortic parameters were assessed in preoperative and postoperative CTAs using 3-dimentional (3D) multiplanar reconstructions (Fig. 2A-C). Preoperative mean transvalvular gradients were available in 12/32 patients.

### Operative technique

All patients underwent surgery using the full root implantation technique, described by us thoroughly in previous literature [8]. In summary, aortic root replacement was performed via median sternotomy on cardiopulmonary bypass and in circulatory arrest. Cardiopulmonary bypass was achieved by cannulation of the ascending aorta and 2-stage venous cannulation. In case of an involvement of the aortic arch or an aortic dissection, arterial cannulation was achieved via the right subclavian artery with the use of an 8 mm vascular graft (*n* = 5). Cardioplegic arrest was enabled with cold crystalloid cardioplegia. After complete transection of the aorta, the aortic valve was resected. Implantation of the freestyle prosthesis was performed with pledgeted sutures. The coronary buttons were reimplanted into neo-ostia using continuous 5–0 polyprolene sutures. The bio-root was anastomosed either to the native aorta or, in the case of ascending aortic treatment, to the vascular graft. If further concomitant surgery was necessary, it was performed in a standard manner.

### Follow up

Echocardiographic examinations were performed according to guidelines before discharge and within the first 6–12 months after surgery [5]. Thereafter, clinical assessment and echocardiography were recommended at annual intervals. CTA FU examinations were performed only for certain indications, such as aortic pathology in the downstream aorta, aortic dissection, or suspected valve dysfunction.

### Imaging analysis

**Fig. 3:**
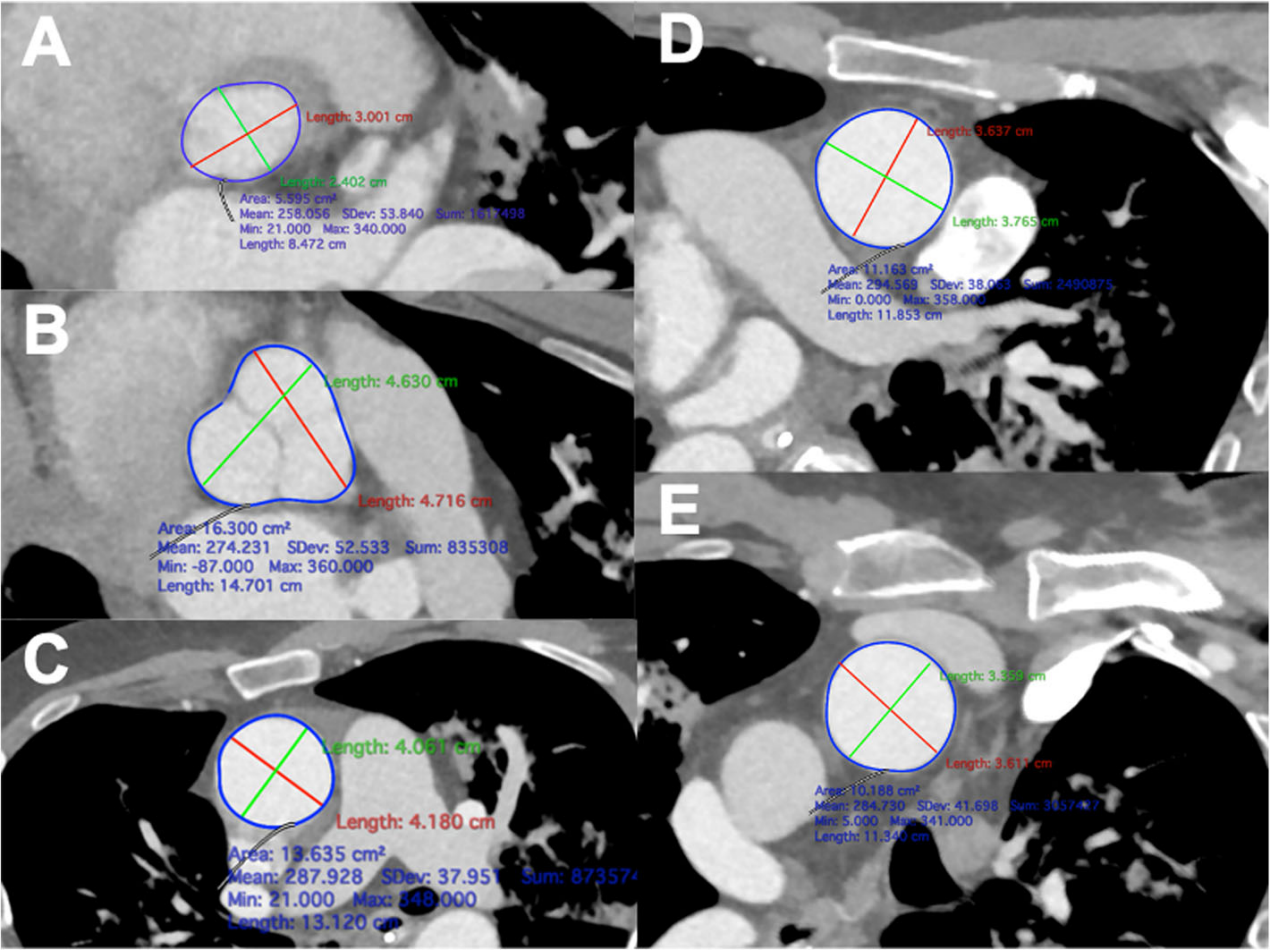
The maximum and minimum diameter and areas of the aortic annulus (**A**), sinus valsalva (**B**), sinotubular junction (**C**), ascending aorta (**D**) and the proximal aortic arch (**E**) were measured using 3D multiplanar reconstruction

The CTA datasets were analyzed in Horos® (Nimble Co LLC d/b/a Purview in Annapolis, MD USA. 4 Version 3.3). Using 3D multiplanar reconstruction, the maximum and minimum diameter (mm) and aortic area (cm^2^) were measured in double-oblique planes at the level of the aortic annulus, sinus valsalva, sinotubular junction (STJ), proximal ascending aorta, and directly proximal to the origin of the brachiocephalic trunk (Fig. 3). A centerline was created from the aortic annulus to the most distal available part of the descending aorta to evaluate the length between the annulus and the aortic arch. Volumetric measurements of the aortic root and ascending aorta were obtained by manual segmentation and subsequent creation of a 3-dimentional model to enable automatic computation of the aortic volume.

### Statistical analysis

Statistical analysis was performed using SPSS (IBM SPSS, Version 27.0. Armonk, NY). Continuous and categorical variables were expressed as mean ± standard deviation or median and percentages, respectively. Normal assumption of continuous variables was validated using the Shapiro–Wilk test. Continuous variables were compared using the paired Student-t test. Correlations between continuous variables were calculated by Pearson’s test (Pearson’s coefficient r). All statistical tests were two sided with the alpha level set at 0.05 for statistical significance.

## Results

### Clinical characteristics

Preoperative patient characteristics of the entire cohort (*n* = 32) are displayed in Table 1. In the majority of patients, aortic root replacement was performed in either an elective (*n* = 19) or urgent (*n* = 8) setting. The indications were aortic regurgitation (*n* = 16), aortic stenosis (*n* = 12), or combined aortic valve pathology (*n* = 7), in combination with dilation of the aortic root (*n* = 14), ascending aorta (*n* = 18), or chronic aortic dissection (*n* = 4). Further indications were aortic valve endocarditis (*n* = 6) or double valve endocarditis (*n* = 3) with abscess formation (*n* = 5). All patients presented with multiple comorbidities, and the median EuroScore II was 7.4% [IQR: 4.46–22.75].

**Table 1:** Patient characteristics

Characteristics	Value
No. of patients	32
**Demographics**	
Age (years)	64.6 ± 10.6
Gender	
Male	22 (68.7%)
Female	10 (31.2%)
Body Mass Index (kg/m^2^)	26.89 ± 5.89
Body Surface Area (cm^2^)	1.98 ± 0.26
**Clinical characteristics**	
Arterial hypertension	30 (93.7%)
Pulmonary hypertension	7 (21.9%)
Chronic obstructive pulmonary disease	8 (25.0%)
NYHA classification	
I	12 (37.5%)
II	7 (21.9%)
III	10 (31.2%)
IV	3 (9.4%)
Diabetes melltitus	8 (25.0%)
Hyperlipoproteinemia	19 (59.4%)
Chronic renal failure	12 (37.5%)
Peripheral vascular disease	5 (15.6%)
Cerebrovascular disease	8 (25.0%)
Previous stroke	4 (12.5%)
Previous cardiac surgery	10 (31.2%)
AVR	5 (15.6%)
AVR with ascending aorta replacement	3 (9.4%)
CABG	1 (3.1%)
CABG, MVR	1 (3.1%)
Previous PCI	4 (12.5%)
Previous endocarditis	4 (12.5%)
EuroSCORE II (%)	7.24 (4.46–22.75)
**Echocardiographic findings**	
Left ventricular ejection fraction (%)	50 ± 13
Aortic insufficiency	
Mild	5 (15.6%)
Moderate	7 (21.9%)
Severe	8 (25.0%)
Aortic stenosis	
Mild	1 (3.1%)
Moderate	2 (6.25%)
Severe	12 (37.5%)
Biscuspid valve	9 (28.1%)
Mitral valve insufficiency	21 (65.6%)
Tricuspid valve insufficiency	14 (43.7%)

*AVR* aortic valve replacement, *CABG* Coronary artery bypass grafting, *PCI* percutaneous coronary intervention

Aortic root replacement in emergency settings (5/32) were performed due to aortic regurgitation (n = 3), endocarditis (*n* = 2), aortic dissection (n = 2), or a combination of the above. Ten patients (31.2%) had a history of previous sternotomy, with eight (25.0%) being for AVR. Preoperative median aortic transvalvular gradient was 23.5 mmHg [IQR: 10.25–41].

### Operative details and clinical outcomes

Concomitant procedures were necessary in 28 out of 32 patients (87.5%), including ascending aorta replacement, hemi arch replacement, additional valve surgery, and coronary artery bypass grafting (Table 2). Mean cardiopulmonary bypass time was 174 ± 87 min. Sizes of the implanted valve varied from 21 mm to 29 mm (mean 26.2 ± 2.4 mm), resulting in a mean effective orifice area of 1.19 ± 0.20 cm^2^.

**Table 2:** Operative details and clinical outcome

Operative details	Value
Cardiopulmonary bypass time (min)	174 ± 87
Crossclamp time (min)	119 ± 53
Valve Size (mm)	26.2 ± 2.4
Effective orifice area (cm^2^)	1.19 ± 0.20
**Concomittant procedure**	
Ascending aorta replacement	21 (65.6%)
Aortic hemi-arch replacement	6 (18.7%)
Mitral valve surgery	
replacement	3 (9.4%)
repair	1 (3.1%)
Tricuspid valve repair	2 (6.25%)
Coronary artery bypass grafting	12 (37.5%)
**Mechanical circulatory support**	
Extracorporeal life support	4 (12.5%)
Intra-aortic balloon pump	3 (9.4%)
**Postoperative Outcomes**	
Bleeding	6 (18.7%)
Thromboembolic event	
Stroke	3 (9.4%)
Myocardial infarction	1 (3.1%)
Postoperative atrial fibrillation	9 (28.1%)
Permanent pacemaker implantation	4 (12.5%)
Dialysis	11 (34.4%)
Sepsis	5 (15.6%)
Prolonged ventilation	12 (37.5%)
Re-intubation	2 (6.25%)
Sternal wound infection	1 (3.1%)
In-hospital mortality	6 (18.7%)

In-hospital mortality was 18.7%. Seven patients required temporary mechanical support in terms of an extracorporeal life support (12.5%) or an intra-aortic balloon pump (9.4%). Six patients (18.7%) underwent re-sternotomy due to bleeding complications. Neurological complications in terms of procedure-related stroke occurred in three patients (9.4%). Postoperative median aortic transvalvular gradient was 5.0 mmHg [IQR: 3.5–13.5].

### Morphological findings

Preoperative minimum and maximum diameter at the level of the aortic annulus were 26.1 ± 3.2 mm and 28.1 ± 3.5 mm, respectively, with a measured aortic area of 6.2 ± 1.5 cm^2^ (Table 3). The implanted valve size (mean 26.2 ± 2.4 mm) and the calculated circular area (πr^2^, 5.4 ± 1.0 cm^2^) strongly correlated with the preoperative minimum and maximum diameter and aortic annulus area (r = 0.78, r = 0.85, r = 0.77, respectively, *p* < 0.001).

**Table 3:** Pre- and postoperative measurements of the aortic root and ascending aorta

	Preoperative (n = 32)	Postoperative (n = 10)	***P*** value*
Aortic annulus			
Diameter max. (mm)	28.1 ± 3.5	26.8 ± 2.9	0.485
Diameter min. (mm)	26.2 ± 3.2	26.3 ± 3.0	0.049
Area (cm^2^)	6.2 ± 1.5	5.8 ± 1.5	0.025
Sinus of Valsalva			
Diameter max. (mm)	40.6 ± 8.6	33.2 ± 6.3	0.092
Diameter min. (mm)	37.1 ± 7.5	30.6 ± 4.9	0.071
Area (cm^2^)	12.5 ± 4.7	5.9 ± 1.5	0.069
Sinotubular junction			
Diameter max. (mm)	39.3 ± 9.8	29.8 ± 4.4	0.021
Diameter min. (mm)	36.0 ± 11.1	28.6 ± 4.1	0.518
Area (cm^2^)	12.1 ± 5.8	6.7 ± 1.8	0.035
Mid-ascending aorta			
Diameter max., (mm)	44.1 ± 9.5	31.8 ± 3.5	0.069
Diameter min. (mm)	42.7 ± 9.5	30.1 ± 2.9	0.070
Area (cm^2^)	15.6 ± 6.3	7.9 ± 1.7	0.070
Proximal aortic arch			
Diameter max. (mm)	37.5 ± 5.9	31.3 ± 4.7	0.087
Diameter min. (mm)	35.1 ± 5.9	29.6 ± 4.9	0.178
Area (cm^2^)	10.6 ± 3.4	8.0 ± 2.4	0.203
Length root, ascending aorta (cm)	10.8 ± 2.2	7.0 ± 1.3	0.005
Volume aortic root, (cm^3^)	45.6 ± 26.3	18.7 ± 4.5	0.029
Volume root and ascending aorta, (cm^3^)	137.3 ± 65.2	54.5 ± 21.1	0.023

*p-value: paired Student-t test (n = 10)

**Fig. 4:**
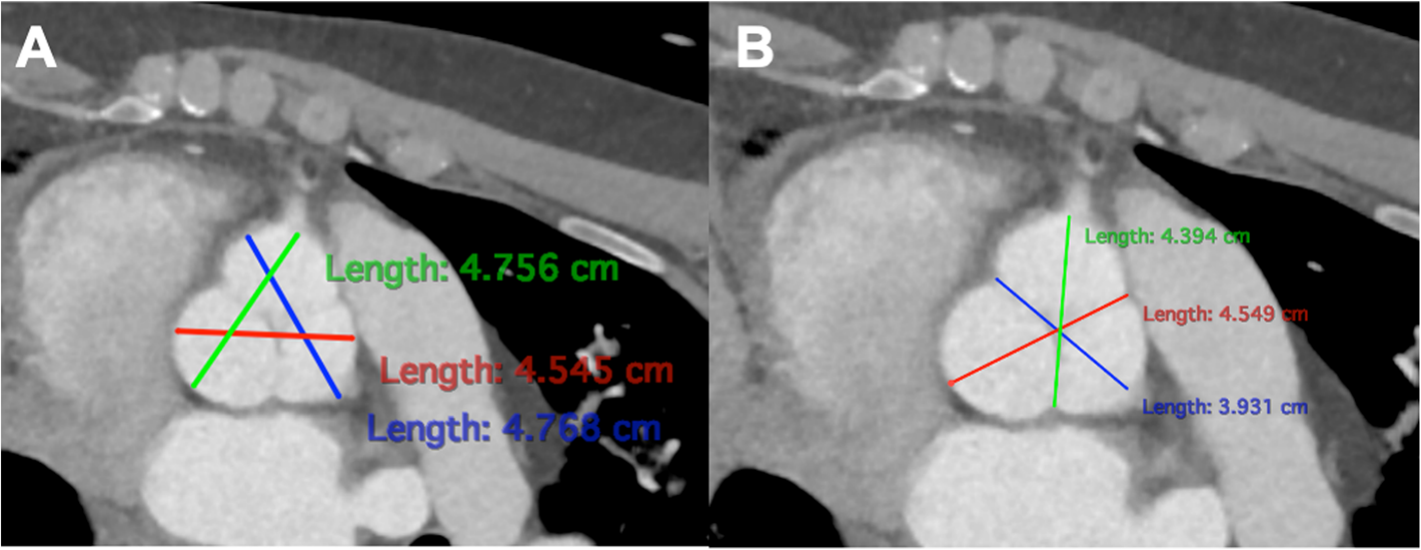
Preoperative measurements of the aortic root were performed sinus-to-sinus (**A**) and sinus-to-commissures (**B**)

The minimum and maximum diameter of the sinus valsalva were 37.12 ± 7.5 mm and 40.59 ± 8.61 mm, respectively. Bicuspid valve was present in 28.1% of the patients. Preoperative measurements of the aortic root were also performed sinus-to-sinus (Fig. 4A) and sinus-to-commissures (Fig. 4B). Mean length of the sinuses of valsalva were 37.9 ± 5.5 mm, 36.7 ± 5.1 mm, 35.3 ± 4.9 mm sinus-to-sinus and 36.8 ± 5.0 mm, 35.3 ± 4.9 mm, 33.7 ± 5.1 mm sinus-to-commissure.

The diagnosis of a root aneurysm varied depending on the use of the minimum or maximum diameter. Five patients had a maximum diameter > 5 cm, and in 12 patients > 4.5 cm, but only two had it > 5 cm and five had it > 4.5 cm when using the minimum diameter. In the mid-ascending aorta, the use of maximum and minimum diameter did not result in a change regarding the diagnosis.

Diameters at the STJ decreased significantly after implantation of the Freestyle prosthesis, but only in terms of the maximum diameter and area. Diameters and areas of the mid-ascending aorta and proximal arch changed depending on the implementation of ascending aortic surgery.

### Volumetric changes

**Fig. 5:**
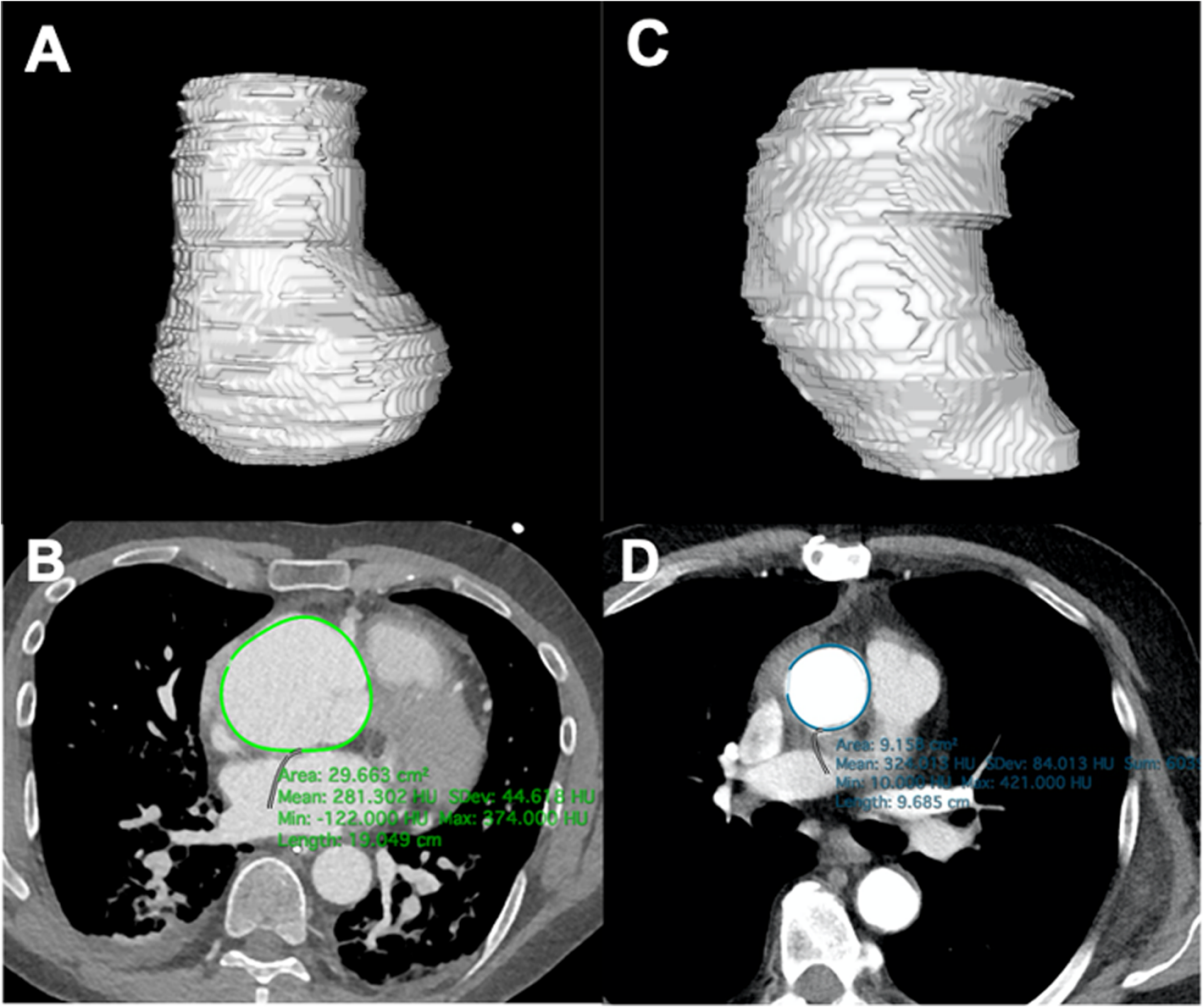
Volumetric measurements were performed on pre- (**A**) and postoperative (**C**) CTAs, with the areas of interest drawn manually in each slice (**B**, **D**). These measurements demonstrate a decrease of the ascending aorta after Freestyle implantation

After Freestyle implantation, with or without ascending or hemi arch replacement, the length from the aortic root to the proximal arch decreased from 10.8 ± 2.2 cm to 7.0 ± 1.3 cm. Volume of the aortic root decreased from 45.6 ± 26.3 cm^3^ to 18.7 ± 4.5 cm^3^ (*p* = 0.029). The combined volume of the aortic root and ascending aorta decreased from 137.3 ± 65.2 cm^3^ to 54.5 ± 21.1 cm^3^ (*p* = 0.023) (Fig. 5).

## Discussion

The Freestyle stentless aortic bioprosthesis has demonstrated excellent long-term clinical and hemodynamic results [9]. The aim of this study was to evaluate the geometrical changes of the aorta following Freestyle prosthesis implantation in the context of clinical outcomes.

The use of a bioprosthesis is recommended in patients over the age of 65 years [5]. The choice between a biological and mechanical valve is also dependent on patient’s preference concerning the trade-off between the potential need for reintervention for valve destruction versus the risk associated with life-long anticoagulation. Further considerations are comorbidities, life expectancy, level of compliance and patient’s lifestyle [10]. If a reintervention becomes necessary, a transcatheter valve-in-valve procedure offers a good option, but entails the implantation of a smaller valve, which may result in patient-prothesis mismatch. Therefore, a mechanical valve is preferable in small annuli [11]. If patients’ characteristics nevertheless require the implantation of a biological valve, a Freestyle prothesis is preferred due to its larger effective orifice area. Furthermore, in case of destructive endocarditis or reoperation, especially if the aortic root tissue is fragile a Freestyle prothesis offers an excellent option.

In our cohort, Freestyle prosthesis was deployed in high-risk patients with infective endocarditis, aortic root enlargement, or aortic dissection. In the vast majority of these patients (87.5%), a concomitant procedure was necessary. Moreover, four patients (12.5%) underwent an isolated root replacement, two of which were redo procedures. One patient suffered from infective endocarditis and the other presented with isolated root dilatation. The population of this particular study group could be explained through the inclusion criteria for performing preoperative CTAs. Furthermore, this study population accounts for an in-hospital mortality of 18.6%, which is elevated when compared to isolated AVR with Freestyle prosthesis. Indeed, in their systematic review Sherrah et al., reported an in-hospital mortality of 5.2% after Freestyle implantation [12]. When our results are placed into perspective, and consideration is made of mortality rates in combined aortic root procedures, high-risk patients, reoperations, or freestyle prosthesis implantation in destructive endocarditis, the outcomes could be considered comparable [13–17].

Following AVR, a high residual transvalvular gradient constitutes a risk factor for worse outcomes, impaired left ventricular diastolic dysfunction, and incomplete regression of left ventricular hypertrophy [18, 19]. The Freestyle aortic bioprosthesis was designed to provide superior hemodynamic performance, more physiological flow patterns, and lower transvalvular gradients [20, 21]. Indeed, a reduction of the mean transvalvular gradient has been described by multiple studies [22]. Yun and colleagues, reported a 41% decrease after AVR with Freestyle prosthesis within the first 6 months, with a corresponding increase in EOA. After 6 months, the gradients remained relatively stable [23]. Accordingly, in our study median transvalvular gradients declined from 23.5 mmHg [IQR: 10.25–41] preoperatively to 5.0 mmHg [IQR: 3.5–13.5] during the postoperative course.

Echocardiographic diagnostics is the gold standard for AVR, evaluating not only the valve itself but also the dimensions of the aorta, which, currently remains the most influential parameter for assessing the risk for aortic dissection and deciding on surgery [24]. As a matter of fact, precise evaluation of the aortic diameter is essential for an accurate diagnosis and further planning of the surgical procedure [25, 26]. Due to the elliptical shape of the aortic annulus, with its maximum diameter lying in the coronary plane, its dimensions can be subject to significant underestimation when using echocardiography or only 2D CT measurements [27]. CT allows for the assessment of the valve anatomy, differentiation between bicuspid and tricuspid valves, and the shape and diameter of the aortic annulus and the left ventricular outflow tract. In our study, the measurements of the aortic annulus strongly correlated with the implanted size of the Freestyle prosthesis. Despite the advantage of sizing under direct vision, accurate pre-operative assessment is important for valve selection and the decision of whether additional surgery is necessary. Especially in patients with asymmetrical aneurysms and bicuspid aortic valve, a significant difference between the minimum and maximum diameter of the aortic root has been described [7]. In the present study, the comparison between the minimum and maximum measured diameter resulted in a more than 20% higher diagnosis rate of root aneurysms > 4.5 cm. On the other hand, 3D volume reconstructions allow the measurement of the entire volume of interest. Therefore, 3D volume measurements are more accurate in detecting small changes in the size of an aneurysm than diameter measurements [28]. Geisbüsch et al. assessed the volume of the ascending aorta in patients with aneurysms compared to a control group [29] and reported a volume of 132.9 ± 39.4 ml in patients with ascending aortic aneurysm and 78.0 ± 24.5 ml in the control group. In our study, the pre-operative volume of the aortic root and ascending aorta were similar (137.27 ± 65.24 cm^3^). Subsequent to Freestyle prosthesis implantation, the length from the aortic root to the proximal aortic arch decreased by approximately 3 cm. Moreover, the diameters and areas decreased to normal values, resulting in a mean volume of 54.5 ± 21.1 cm^3^ [30]. These volumetry results demonstrate an excellent restoration of the aortic root and ascending aortic geometry.

### Study limitations

The presented study is an observational assessment of clinical and morphological outcomes. Patients were not randomly assigned to different therapies. The study is therefore limited by its retrospective, nonrandomized, single-center nature. We observed only patients with Freestyle prothesis, therefore we are not able to generalize the findings for different valve types.

The main limitation of this study is the relatively small cohort size, caused by the inclusion criterion for the availability of preoperative CTAs. Since we do not routinely perform a FU CTA, postoperative CT was not available in all patients.

## Conclusions

Freestyle prosthesis is an excellent option for high-risk patients with concomitant aortic pathology or destruction of the aortic root due to endocarditis. CTA measurements provide substantial information for surgical planning and therefore, if available, should be considered in pre-operative planning.

## Data Availability

The authors declare that all data supporting the findings of this study are available within the article.

## Abbreviations

3D: 3-dimentional
AVR: Aortic valve replacement
CTA: Computed tomography angiography
IQR: Interquartile range
STJ: Sinotubular junction
